# Evolution of alternative and constitutive regions of mammalian 5'UTRs

**DOI:** 10.1186/1471-2164-10-162

**Published:** 2009-04-16

**Authors:** Alissa M Resch, Aleksey Y Ogurtsov, Igor B Rogozin, Svetlana A Shabalina, Eugene V Koonin

**Affiliations:** 1 National Center for Biotechnology Information, National Library of Medicine, National Institutes of Health, Bethesda, Maryland 20894, USA

## Abstract

**Background:**

Alternative splicing (AS) in protein-coding sequences has emerged as an important mechanism of regulation and diversification of animal gene function. By contrast, the extent and roles of alternative events including AS and alternative transcription initiation (ATI) within the 5'-untranslated regions (5'UTRs) of mammalian genes are not well characterized.

**Results:**

We evaluated the abundance, conservation and evolution of putative regulatory control elements, namely, upstream start codons (uAUGs) and open reading frames (uORFs), in the 5'UTRs of human and mouse genes impacted by alternative events. For genes with alternative 5'UTRs, the fraction of alternative sequences (those present in a subset of the transcripts) is much greater than that in the corresponding coding sequence, conceivably, because 5'UTRs are not bound by constraints on protein structure that limit AS in coding regions. Alternative regions of mammalian 5'UTRs evolve faster and are subject to a weaker purifying selection than constitutive portions. This relatively weak selection results in over-abundance of uAUGs and uORFs in the alternative regions of 5'UTRs compared to constitutive regions. Nevertheless, even in alternative regions, uORFs evolve under a stronger selection than the rest of the sequences, indicating that some of the uORFs are conserved regulatory elements; some of the non-conserved uORFs could be involved in species-specific regulation.

**Conclusion:**

The findings on the evolution and selection in alternative and constitutive regions presented here are consistent with the hypothesis that alternative events, namely, AS and ATI, in 5'UTRs of mammalian genes are likely to contribute to the regulation of translation.

## Background

Alternative splicing (AS) has emerged as a major mechanism for regulating gene expression and function in animals, particularly, in mammals. Large-scale studies based on mapping of expressed sequence data on genomic sequences have produced estimates of as many as 30–60% of human genes undergoing alternative splicing [1-5]. The impact of alternative splicing on protein function has been studied in great detail and is generally recognized as a source of protein diversity that expands the repertoire of protein function [6-8].

In contrast, little is known about the prevalence and impact of alternative events, such as AS and alternative transcription initiation (ATI), in 5'-untranslated regions (5'UTRs). AS and ATI are the primary sources of 5'UTR transcript diversity, and several reports have conjectured that these mechanisms might play an important role in orchestrating complex regulatory mechanisms within the 5'UTRs [9-13]. Estimates of the number of genes with alternative 5'UTRs vary from 12% [14] to 22% [5], while estimates of alternative promoter usage range from 10% [15] to 18% [16]. Anecdotally, studies have shown that alternative events are responsible for 5'UTR transcript diversity in mammals, but to our knowledge, there have been no detailed, genome-wide studies aimed at elucidating the functional role of transcript diversity in mammalian 5'UTRs.

The bias toward studying AS in the coding regions versus 5'UTR probably reflects two obvious sources of complications in the analysis of transcript diversity in UTRs. First, it is easier to assess the functional impact of AS in protein-coding regions because elimination or disruption of known protein domains resulting from AS is readily interpretable [6-8]. Second, boundaries of the coding sequence typically are identified with relative ease because protein sequences are defined by their start and stop codons whereas precise delineation of the 5'UTR often is problematic.

Translational efficiency of eukaryotic mRNAs depends on the presence of regulatory elements within the 5'UTR. It has been shown that the occurrence of initiation codons and open reading frames upstream of the authentic start codon (uAUGs and uORFs, respectively) can affect the translation of mRNA into protein. The presence of uAUGs and uORFs in mammalian 5'UTRs is typically associated with translational repression [10,17] but cases of increased translation efficiency also have been reported [18]. A complementary computational study has demonstrated substantial conservation of uAUGs and uORFs in 5'UTRs of mammalian mRNAs, suggesting that at least some of these elements are functionally important [19]. These experimental and computational findings raise the possibility that 5'UTR diversity has the potential to produce mRNA isoforms that differ with respect to their uAUG and uORF content, which could be an important facet of the regulation of translation.

Experimental evidence shows that 5'UTR transcript diversity is achieved during transcription, via ATI, and after transcription, via AS. In some instances, both mechanisms are employed. For example, AS in the 5'UTR of human axin2, a negative regulator of Wnt/B-catenin signaling, generates three isoforms with different arrangements of uAUGs and uORFs, resulting in a set of 5'UTRs that each confer different mRNA stabilities and translational efficiencies upon the respective isoforms [20]. Similarly, an alternatively spliced exon located in the 5'UTR of neuronal nitric-oxide synthase (nNOS) has been shown to introduce a translational control element that inhibits translation of the mRNA [21]. The diversity and complexity of the mu-opioid receptor gene expression is achieved by a combination of alternative splicing and alternative promoter usage [11]. Translational repression of the mouse mu-opioid receptor expression using uORFs and leaky scanning has been recently reported [17].

The significance of the ATI and AS mechanisms in generating 5'UTR transcript diversity lies with the ability to alter the 5'UTR landscape by rearranging both the number and type of translational control elements included in each transcript. Slight differences in the arrangement of translational control elements between isoforms can lead to major changes in regulatory effects on translation. For instance, translational regulation of the multidrug resistance-associated protein 2 (Mrp2) is mediated by ATI. The 5'UTR of Mrp2 contains four different transcription start sites and three uORFs, and experimental data show that uORF3 has a much stronger inhibitory effect on translation than uORF1 and uORF2 [13]. A combination of ATI and AS in Dicer, a ribonuclease that mediates RNA interference at the transcriptional and post-transcriptional levels, appears to regulate translational efficiency as well, resulting in long and short transcript variants. Both variants encode uAUGs (9 in the long form and five in the short form), and although both forms show decreased levels of translation, the longer form appears to exhibit greater inhibitory effects, probably, because of the increased number of uAUGs [9]. Likewise, Tie2, an endothelium-specific receptor tyrosine kinase required for blood vessel maturation, contains multiple transcription start sites and encodes five uORFs. Apparently, the greater the number of uORFs contained in the 5'UTR, the greater the inhibitory effects on translation, suggesting an accumulative effect on the overall level of translational efficiency [12].

In addition, it has been shown that both evolutionarily conserved and non-conserved regulatory control elements exhibit inhibitory effects, suggesting that non-conserved control elements may regulate at a species-specific level. For example, human oncogene mdm2 contains two uORFs, both conserved in mouse. Although the inhibitory effect of uORF1 exceeds that of uORF2, both uORFs are required for maximum inhibition of translation [22]. In contrast, the human stress response transcription factor ATF5 contains two uORFs that are conserved in mouse, as well as three non-conserved species-specific uAUGs. The alternative transcript encoding the conserved uORFs exhibits a stronger inhibitory effect than the transcript encoding the non-conserved uAUGs, but the effect of the non-conserved elements is non-negligible as well [23,24].

We performed a genome-wide comparative study of 5'UTR sequences in primates and rodents with the principal goal of understanding how alternative events impact regulation of translation. To this end, we compared the abundance, conservation and evolution of translational control elements within alternative and constitutive regions of 5'UTR. Alternative nucleotides were not classified according to type of alternative event, because the focus of the study was to examine the broad impact of transcript diversity on mammalian 5'UTRs. Accordingly, we reasoned that the regulatory effects of uAUGs and uORFs located in alternative regions of 5'UTRs should be the same regardless of whether the existing transcript diversity for the given gene is generated via ATI, AS, or both. We find that, although alternative regions of mammalian 5'UTRs evolve faster and are subject to a weaker purifying selection than constitutive regions, they possess extensive potential for translation regulation.

## Results and discussion

### 5'UTR statistics for human and mouse

A genome-wide comparative analysis of 5'UTRs in human and mouse was carried out to investigate the prevalence, conservation, and evolution of putative translational control elements within alternative and constitutive regions of mammalian 5'UTRs. Starting with high quality annotation from the ASD Database, we restricted our analysis of alternative events to those that involved exclusively 5'UTR sequences, and limited the pool of alternative transcript data to reliably identified, high quality isoforms with mRNA evidence and known protein-coding sequences (see Methods for details). A total of 7735 human isoforms (with 5'UTR sequences) were used to identify 2915 human genes with alternative 5'UTRs, and 2165 mouse isoforms (also with 5'UTR sequences) were similarly used to identify a set of 909 mouse genes with alternative 5'UTRs (hereinafter ALT_5'UTR sets). These stringent criteria likely yield a relatively small subset of the complete set of genes with alternative 5'UTRs, but were chosen to eliminate potentially unreliable splice predictions and alignment artifacts from biasing the results. Our ALT_5'UTR subsets represent 12% of human genes and 3.4% of mouse genes. Previous reports suggest that 10–22% of human genes [5,14,15] and 19–20% of mouse genes [25,26] contain alternative 5'UTRs. Our estimates for mouse are lower than those previously reported [25,26]; most likely, these low values reflect the fact that there is less high quality annotation for alternative events in 5' UTRs available for mouse than there is for human. Our estimates, which are based on mRNA evidence, probably, represent the lower bounds for the number of mammalian genes with ALT_5'UTRs.

Are 5'UTR lengths distributed differently between genes that exhibit 5'UTR transcript diversity versus those that do not? We address this question by identifying a set of control genes that do not contain alternative events (hereinafter referred to as the nonALT control sets). Comparison of the 5'UTR length distributions between transcripts from the ALT and nonALT sets reveals that, in humans, 5'UTR lengths are distributed differently (Additional file 1). The human ALT 5'UTRs are, on average, slightly but significantly longer than nonALT 5'UTRs, whereas, in mouse, 5'UTR lengths are approximately the same between the ALT and nonALT sets (Table 1). The 5'UTR lengths reported here are close to the generally accepted range of 160–210 nucleotides for human [27,28], and just above the estimate of 139 nucleotides for mammals [29]. The origin and significance of the length difference between ALT and nonALT 5'UTRs in humans but, apparently, not in mouse remain unclear. A distinct possibility seems to be that the length of mouse ALT 5'UTRs is underestimated owing to the insufficient availability of sequences of low-abundance isoforms. Should the greater length of the human ALT 5'UTRs compared to nonALT be taken as an accurate representation of the relationship in mammals, it is likely to reflect the greater opportunity for alternative events in longer 5'UTRs.

**Table 1:** 5'UTR statistics for ALT and nonALT gene sets

	**Human**		**Mouse**	
	**nonALT**	**ALT**	**nonALT**	**ALT**
Total genes	11,727	2,915	14,288	909

5'UTR length	203	239.6	180.1	178.1

Genes with uORFs	5212 (44%)	1547 (53%)	5968 (42%)	460 (51%)
uORF length	58.7	73.4 (ALT)	54.1	63.1 (ALT)
		48.2 (CONSTIT)		44 (CONSTIT)

Genes in the ALT_5'UTR set exhibit 5'UTR transcript diversity (AS and ATI), whereas, genes in the nonALT control set do not. Total number of genes in each set (top tier). Average 5'UTR length (nts) for transcripts in each set (middle tier). Total number of genes that contain uORFs (percentage in parenthesis), and average uORF lengths (nts) in alternative (ALT) and constitutive (CONSTIT) regions (bottom tier).

### Higher prevalence of alternative sequences in 5'UTRs compared to coding regions

**Figure 1:**
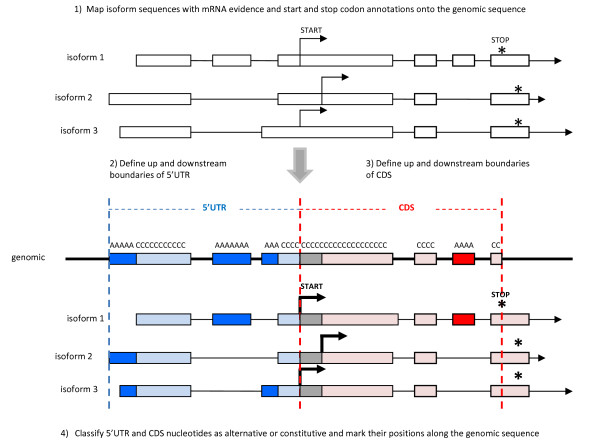
**Classification of alternative and constitutive nucleotides**. The procedure to classify nucleotides as alternative or constitutive is outlined in steps 1–4. Start and stop codons are marked by arrows and asterisks, respectively. Alternative regions of 5'UTR and CDS are colored dark blue and dark red, respectively, whereas constitutive regions are colored light blue and light red. Variable regions (those classified as alternative or constitutive, depending on the isoform) are colored gray. Alternative nucleotide positions are labeled "A" along the genomic sequence, while constitutive nucleotide positions are labeled "C".

To assess the prevalence of alternative events within mammalian 5'UTRs, we classified 5'UTR nucleotides as ALT or CONSTIT by examining nucleotide inclusion levels within the pool of alternative transcripts for each gene. CONSTIT nucleotides are present in all isoforms, whereas ALT nucleotides are present only in a fraction of the isoforms (Figure 1). Alternative isoforms were mapped to the genomic sequence, classified as 5'UTR or CDS (based on the 5'UTR and CDS boundaries defined in Methods and Figure 1), and then annotated as either ALT or CONSTIT. Among human genes, we counted 189,778 CONSTIT and 548,760 ALT nucleotides in the 5'UTRs included in the present analysis. For purposes of comparison, we counted 2,984,181 CONSTIT and 1,072,755 ALT nucleotides in the corresponding coding sequences of the same genes (Table 2). Thus, the ratio of alternative-to-constitutive nucleotides is reversed in the 5'UTRs compared to the CDS: the average of alternative-to-constitutive nucleotides was ~3:1 in the 5'UTR, and ~1:3 in the CDS. A qualitatively similar pattern was observed in mouse, where estimates show an average ratio of ~2:1 between alternative-to-constitutive nucleotides in the 5'UTR, and ~1:4 in the CDS of the analyzed genes (Table 2). The CDS boundaries chosen for this calculation include the variable region located between the most upstream and downstream start codons (Figure 1). We define this stretch of genomic sequence as variable because it is included as 5'UTR in some isoforms, but as CDS in others. If we remove this variable region from the CDS altogether and define the upstream CDS boundary by the most downstream start codon, we estimate an average ratio of ~1:9 between alternative-to-constitutive nucleotides in the human CDS, and a ~1:12 ratio between alternative-to-constitutive nucleotides in the mouse CDS. A variable region also exists at the CDS|3'UTR boundary, but this region was removed from our analysis by simply choosing the most upstream stop codon as the downstream CDS boundary (Figure 1). These results indicate that, among genes with alternative 5'UTRs, the extent of alternative events in the 5'UTRs is much greater than that in the coding regions.

**Table 2:** Number of alternative and constitutive nucleotides in 5'UTR and CDS

**Human**	**ALT**	**CONSTIT**	**ALT:CONSTIT ratio**
5'UTR	548,760	189,778	3:1
CDS	1,072,755	2,984,181	1:3

**Mouse**	**ALT**	**CONSTIT**	**ALT:CONSTIT ratio**

5'UTR	132,729	59,421	2:1
CDS	252,539	1,105,911	1:4

Number of alternative (ALT) and constitutive (CONSTIT) nucleotides in the 5'UTR and CDS regions of genes in the ALT_5'UTR sets for human and mouse. Ratio of alternative-to-constitutive nucleotides is shown in the column on the right.

### Excess of potential regulatory elements in alternative regions of 5'UTR

Given that the fraction of alternative sequence is greater in the 5'UTRs than in the coding region among genes with alternative 5'UTR events, it is natural to ask how often 5'UTRs from this set harbor putative control elements that might be involved in translation regulation. We examined the organization of putative regulatory motifs within ALT and CONSTIT regions of 5'UTR by searching for potential regulatory elements including uAUGs and uORFs.

Previously, uAUGs have been detected in 29–48% of mammalian 5'UTRs [30-33], and anecdotally, often have been found in alternatively spliced genes [34,35]. We compared the frequency of uAUGs between ALT and CONSTIT regions of 5'UTR to see if the 5'UTR transcript diversity contributes to the overall levels of uAUG abundance. Raw counts indicate that uAUGs are 3.9 times more frequent in ALT versus CONSTIT regions in human and 2.7 times more frequent in mouse (Table 3).

**Table 3:** Frequency of uAUGs and uORFs in alternative and constitutive regions

	**Human**	**Mouse**
Total uAUGs mapped to genomic	6228	1517
uAUGs mapped to ALT	4948 (79%)	1108 (73%)
uAUGs mapped to CONSTIT	1280 (21%)	409 (27%)
uAUGs included in evolutionary conservation analysis in Human-Macaque and Mouse-Rat orthologs	5608	1518

Total uORFs mapped to genomic	4870	1255
uORFs mapped to ALT	3807 (78%)	906 (72%)
uORFs mapped to CONSTIT	1063 (22%)	349 (28%)
uORFs fully contained within ALT	3042 (80%)	667 (74%)
uORFs fully contained within CONSTIT	901 (85%)	324 (94%)
uORFs included in evolutionary conservation analysis in Human-Macaque and Mouse-Rat orthologs	1988	842

Count of uAUGs (top tier) and uORFs (bottom tier) that map to alternative (ALT) and constitutive (CONSTIT) regions of 5'UTR in human and mouse (percentages in parenthesis).

We compared the relative frequency of all 64 codons within ALT and CONSTIT regions of 5'UTR and, in accord with previous observations [19], found that the AUG codon is significantly depleted in both regions (Additional file 1). In addition, we compared the frequency of AUG with those of the 5 triplets that represent permutations of AUG (AGU, GUA, GAU, UAG and UGA), and again observed significant depletion of AUG in both ALT and CONSTIT regions in human and mouse (Table 4). Thus, there seems to be purifying selection against uAUGs in both alternative and constitutive regions of mammalian 5'UTRs, but this selection appears to be substantially weaker in alternative regions, resulting in the observed higher frequency of AUG.

**Table 4:** Observed and expected frequency of AUG triplets and shuffled triplets

		**Human**		**Mouse**	
		**Obs**	**Exp**	**Obs**	**Exp**

ATG	ALT	10.2	12.9	10.4	13
	CONSTIT	7.9	12.4	8.6	12.7

AGT/GTA/GAT/TAG/TGA	ALT	60.9	64.5	65.8	65.0
		(12.1)	(12.9)	(13.2)	(13.0)
	CONSTIT	58.7	62.0	63.7	63.5
		(11.7)	(12.4)	(12.8)	(12.7)

Observed and expected frequencies of AUG triplets (top tier) and shuffled triplets (AGT/GTA/GAT/TAG/TGA) that are permutations of AUG (bottom tier), per 1000 nucleotides in ALT and CONSTIT regions of 5'UTR. The significance of the differences between expected and observed frequencies of uAUG and shuffled triplets was estimated using the χ^2 ^test. All comparisons using the χ^2 ^test are highly significant (P < 10^-10^) except for two cases in mouse: observed ALT_AGT/GTA/GAT/TAG/TGA versus expected ALT_AGT/GTA/GAT/TAG/TGA (P = 0.41) and observed CONSTIT_AGT/GTA/GAT/TAG/TGA versus expected CONSTIT_AGT/GTA/GAT/TAG/TGA (P = 0.97).

The efficiency of translation initiation depends on the arrangement of nucleotides surrounding the translational start codon, so the AUG context is thought to be an important regulatory factor (reviewed in [36]). The nucleotide contexts of uAUGs located in ALT and CONSTIT regions were evaluated using previously published methods [28], and no significant differences were detected in either human or mouse. The majority of uAUGs in ALT and CONSTIT regions exhibited weak contexts (Additional file 2), consistent with previous results [19,28] and with the above conclusion on selection against AUGs in 5'UTRs, in that uAUGs are unlikely to efficiently initiate translation.

Next, we estimated the size and abundance of uORFs in ALT and CONSTIT regions of 5'UTR in order to assess the role of uORFs as potential control elements. The uORFs were identified in the 5'UTR sequences of transcripts, mapped to the genomic sequence, and then classified as ALT or CONSTIT using the same approach that was applied to the mapping of uAUGs. To be included in the analysis, a uORF must be fully contained in either the ALT or CONSTIT regions of the corresponding 5'UTR (uORFs that spun the boundaries between ALT and CONSTIT regions were removed from the final data set). The great majority of uORFs (81% and 79% in mouse) indeed are fully contained within ALT or CONSTIT regions, indicating that restricting the analysis to these ORFs would not significantly bias the results.

**Figure 2:**
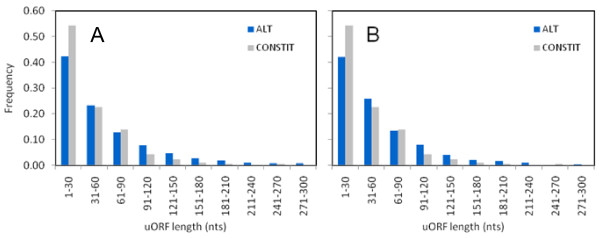
**uORF length distributions in alternative and constitutive regions**. uORF length distributions between alternative (ALT) and constitutive (CONSTIT) regions of 5'UTR are significantly different in human (A) and mouse (B) (P = 1.1 × 10^-26 ^for human and P = 6.5 × 10^-9 ^for mouse; Student's t-test). uORFs in ALT and CONSTIT regions are labeled as blue and gray bars in histograms.

The alternative regions of 5'UTRs contained 3.6 times more uORFs than constitutive regions in humans and 2.6 times more uORFs in mouse (Table 3). A comparison of the uORF length distributions between ALT and CONSTIT regions of 5'UTR showed that ALT uORFs are significantly longer than CONSTIT uORFs (P = 1.1 × 10^-26 ^for human and P = 6.5 × 10^-9 ^for mouse; Student's t-test) (Figure 2 and Table 1). To control for length differences between ALT and CONSTIT regions of 5'UTR, we compared uORF length distributions between size-matched ALT and CONSTIT regions for a subset of 320 human genes taken from the original ALT_5'UTR set. The results indicate that after controlling for length, ALT uORFs are still markedly and significantly longer than CONSTIT uORFs (72.9 versus 48.6 nucleotides; P = 0.00006; Student's t-test; Additional file 1).

We further addressed the question whether uORFs are more prevalent in 5'UTRs of genes that contain alternative regions? Comparison of uORF abundance between genes in the ALT and nonALT control sets indicates that ~53% of human and ~51% of mouse ALT genes contain uORFs, whereas only ~44% of human and ~42% of mouse nonALT control genes contain uORFs, suggesting that uORFs are indeed more common in 5'UTRs containing alternative regions (Table 1). Comparison of uORF densities (the number of uORFs per gene) between genes from the ALT and nonALT control sets showed substantially greater densities in the ALT sets in both human (P = 1.46 × 10^-11^; Student's t-test) and mouse (P = 0.0004; Student's t-test) (Additional file 1). Genes from the ALT set contain an average of 3.1 and 2.7 uORFs per gene in human and mouse, respectively, whereas genes from the nonALT control set contain an average of 2.4 uORFs per gene in human and 2.2 in mouse. A comparison of uORF length distributions between the ALT and nonALT sets showed that uORFs from the nonALT control set are significantly longer than uORFs located in constitutive regions of ALT genes, but shorter than uORFs located in alternative regions of ALT genes (Table 1 and Additional file 1). Taken together, these observations show that genes with alternative 5'UTRs are more likely to encode uORFs than genes without such regions, and that uORFs are more abundant in alternative than in constitutive regions. The results obtained for uORFs are fully consistent with those for the uAUGs and suggest that there is a weaker selection against translation initiation upstream of the authentic AUG in alternative regions of mammalian 5'UTRs than there is in constitutive regions. From a complementary perspective, one would note that alternative regions contain a greater concentration of potential control elements that could regulate translation.

We examined the nucleotide composition of human and mouse 5'UTRs and found that GC-contents for human and mouse 5'UTRs were estimated, respectively, at 60% and 59% (Additional file 2), in good agreement with previous reports [27,28]. No significant differences in nucleotide composition or GC-content were detected between ALT and CONSTIT regions of the 5'UTR. Thus, the excess of uORFs in alternative versus constitutive regions of 5'UTRs is not caused by differences in nucleotide compositions of these regions.

### Conservation of putative control elements in constitutive and alternative regions of mammalian 5'UTRs

The evolutionary conservation of uAUGs and uORFs in ALT and CONSTIT regions of mammalian 5'UTRs was evaluated using alignments of orthologous sequences in two pairs of closely related species, namely, human-macaque and mouse-rat. In general, and in agreement with previous observations [19], the uAUGs were found to be highly conserved, which is consistent with their widespread regulatory roles. Between human and macaque, 81% of the CONSTIT uAUGs are conserved as compared to 74% of the ALT uAUGs, a relatively small but statistically significant difference (P = 0.000002; Fisher's exact test) (Table 5). A similar pattern was observed for the mouse-rat comparison: 70% of CONSTIT uAUGs and 59% of ALT uAUGs are conserved (P = 0.00007; Fisher's exact test). We also compared the frequency of conserved AUGs to the frequency of the five shuffled triplets that are permutations of AUG, and found that the AUGs are significantly more conserved than the other five triplets within CONSTIT regions of 5'UTR in human (P = 3.7 × 10^-6^) and mouse (P = 8.2 × 10^-6^). The fraction of conserved AUGs in ALT regions was slightly, but significantly greater than the fraction of the other five triplets in human (P = 0.05), but not in mouse (P = 0.21) (Table 5). Thus, the AUGs in CONSTIT and ALT regions are significantly conserved compared to the background levels of conservation, but that conservation is considerably more pronounced in CONSTIT regions.

**Table 5:** Conservation of uAUGs and uORFs in alternative and constitutive regions

		**Hum-Mac**		**Mm-Rat**	
		**Con**	**NonCon**	**Con**	**NonCon**

ATG	ALT	3245(74%)	1119(26%)	642(59%)	442(41%)
	CONSTIT	1005(81%)	239(19%)	305(70%)	129(30%)

AGT/GTA/GAT/TAG/TGA	ALT	17498(73%)	6493(27%)	3937(57%)	2925(43%)
	CONSTIT	6525(75%)	2190(25%)	1904(59%)	1314(41%)

uORF	ALT	910(61%)	593(39%)	290(41%)	409(59%)
	CONSTIT	348(72%)	137(28%)	81(57%)	62(43%)

Conservation of uAUGs and uORFs in alternative (ALT) and constitutive (CONSTIT) regions of 5'UTR for human-macaque and mouse-rat. Conservation of AUG triplets in ALT and CONSTIT regions (top tier). The significance between conserved (Con) and non-conserved (NonCon) AUG frequencies in ALT and CONSTIT regions was estimated using Fisher's exact test (P = 0.000002 for human-macaque; P = 0.00007 for mouse-rat). Conservation of AGT, GTA, GAT, TAG and TGA shuffled triplets in ALT and CONSTIT regions (middle tier). Fisher's exact test for the fraction of conserved AUG triplets produced significant results in human: P = 0.05 (for ALT AUG versus shuffled triplets); P = 3.7 × 10^-6 ^(for CONSTIT AUG versus shuffled triplets), and in mouse: P = 8.2 × 10^-6 ^(for CONSTIT versus shuffled triplets). Results for mouse ALT AUG versus shuffled triplets were insignificant (P = 0.21). Conservation of uORFs in ALT and CONSTIT regions of 5'UTR (bottom tier). The significance between conserved and non-conserved uORF frequencies in ALT and CONSTIT regions was estimated using Fisher's exact test (P = 6.5 × 10^-9 ^for human-macaque; P = 0.001 for mouse-rat).

Analysis of uORF conservation yielded similar results, i.e., 71.8% of CONSTIT uORFs and 60.5% of ALT uORFs were conserved between human and macaque (P = 6.5 × 10^-9^; Fisher's exact test), and the corresponding values for mouse-rat were 56.6% and 41.5% (P = 0.001; Fisher's exact test) (Table 5). With regard to the substantially lower conservation of uORFs in alternative regions, it is necessary to point out that, although evolutionary conservation is a strong indicator of the functional relevance of the corresponding element, it is not a strict requirement. In particular, both conserved and non-conserved control elements have been implicated in translational repression [22,23,37]; obviously, non-conserved elements are more likely to exert species-specific regulation. For instance, the 5'UTR of the mu-opioid receptor gene contains non-conserved control elements that inhibit translation in a species-specific fashion [38]. Similarly, species-specific patterns of 5'UTR alternative exon usage have been linked to species-specific patterns of tissue expression [39-41].

A recent analysis of uORF conservation in four *Cryptococcus *species has led to the estimate that approximately one-third of the uORFs are conserved owing to their importance for the regulation of translational efficiency [42]. Substantial conservation of uORFs has also been observed in 5'UTRs of *Saccharomyces *[43] and plants [44], findings that are compatible with the possibility that many uORFs are associated with biological functions. Neafsey and Galagan report that the majority of conserved uORFs in *Cryptococcus *do not exhibit codon usage bias or conservation at the amino acid level, effectively ruling out the possibility that a significant fraction of the uORFs encode functional peptides [42]. We performed a similar analysis and found no evidence of codon usage bias among the human and mouse uORF sequences included in this study. We repeated this analysis after partitioning the uORFs according to ALT and CONSTIT in human, and found that, in general, profiles of relative codon frequencies are similar between all codons in ALT and CONSTIT uORFs (Additional file 1). Thus, it appears likely that, to the extent that they are functional, most of the uORFs are control elements, although a minority might encode biologically relevant peptides. Indeed, a recent proteomic analysis resulted in the identification of 54 proteins, less than 100 amino acids in length each, that are suspected of being translated from uORFs located in the 5'UTRs of human mRNAs [45].

### Fast evolution of alternative regions in mammalian 5'UTRs

To further assess the evolutionary forces that affect mammalian 5'UTRs, we compared the evolutionary rates between ALT and CONSTIT regions and found that ALT regions diverge faster than CONSTIT regions in both primates and rodents. Significant differences between the ALT and CONSTIT regions of 5'UTRs were detected for both synonymous (Ks) (P = 0.022 for human-macaque and P = 0.004 for mouse-rat; Wilcoxon rank test) and non-synonymous (Ka) (P < 0.001 for human-macaque and mouse-rat; Wilcoxon rank test) substitution rates in uORFs (Table 6). We calculated non-synonymous (Ka) and synonymous (Ks) substitution rates for uORFs in ALT and CONSTIT regions, and then re-calculated rates for the subset with lengths ≥ 30 nts, to ensure that short uORFs did not bias the results (Table 6). The substitution rates of the uORFs did not significantly change after removal of the short subset from either the ALT or the CONSTIT regions in human-macaque and mouse-rat comparisons. Substitution rates within ALT and CONSTIT portions of 5'UTR were calculated separately for regions that encode uORFs (K_5 uORFs_) and regions that lack uORFs (K_5 uORFs excluded_), and again, ALT regions appear to diverge more rapidly than CONSTIT regions (Table 6). The uORFs in both ALT and CONSTIT regions evolve slower and, by inference, are subject to stronger selection than the rest of the sequence (K_5 uORFs _< K_5 uORFs exluded_) (P = 0.009 for human-macaque and P = 0.04 for mouse-rat; Wilcoxon rank test). Furthermore, there is a weak but significant overall trend between substitution rates, Ka < Ks, in both ALT and CONSTIT regions, suggesting that non-negligible, although weak purifying selection affects the amino acid sequences encoded in uORFs, conceivably, owing to a small fraction of the uORFs that produce functional peptides. Taken together, these observations indicate that ALT regions are subject to a weaker purifying selection than CONSTIT regions, however, a fraction of the uORFs in the ALT regions could represent conserved translational control elements. Because the evolutionary rate distributions for uORFs in ALT and CONSTIT regions substantially overlap, it is impossible to rule out the possibility that there are small subsets of highly conserved alternative uORFs, a potential target for future investigations.

**Table 6:** Substitution rates for alternative and constitutive regions of 5'UTR

**Human-Macaque**		**Ka**	**Ks**	**K_5(uORFs)_**	**K_5(uORFs excluded)_**
ALT	all	0.045 ± 0.001	0.052 ± 0.001	0.047 ± 0.001	0.054 ± 0.001
	≥ 30 bp	0.046 ± 0.001	0.051 ± 0.002		

CONSTIT	all	0.040 ± 0.001	0.046 ± 0.002	0.042 ± 0.001	0.051 ± 0.002
	≥ 30 bp	0.041 ± 0.002	0.048 ± 0.003		

**Mouse-Rat**					
		**Ka**	**Ks**	**K_5(uORFs)_**	**K_5(uORFs excluded)_**

ALT	all	0.092 ± 0.002	0.111 ± 0.002	0.099 ± 0.002	0.098 ± 0.001
	≥ 30 bp	0.094 ± 0.004	0.114 ± 0.005		

CONSTIT	all	0.088 ± 0.003	0.103 ± 0.004	0.093 ± 0.002	0.100 ± 0.001
	≥ 30 bp	0.091 ± 0.004	0.108 ± 0.007		

Substitution rates for alternative and constitutive regions of 5'UTR were estimated for human-macaque (top tier) and mouse-rat (bottom tier). Evolutionary rates for non-synonymous (Ka) and synonymous (Ks) sites from uORFs within ALT and CONSTIT regions were estimated using the Pamilo-Bianchi-Li method. Rates were calculated for all uORFs (all) and for the subset of uORFs ≥ 30 nts in length (≥ 30 bp). Evolutionary rates were also calculated for regions of 5'UTR that contain uORFs (K_5 uORFs_) and for regions without (K_5 uORFs excluded_), using the Kimura-2-Parameter method.

The present analysis indicates that ALT regions of mammalian 5'UTRs evolve faster than CONSTIT regions, in all likelihood, owing to relaxed purifying selection. There is no consensus regarding trends in evolutionary rates between ALT and CONSTIT regions of coding sequence (CDS) [46]. Nevertheless, most comparisons indicate a higher non-synonymous substitution rate (Ka) in alternative exons [47,48], but a significantly lower synonymous substitution rate (Ks) [49], resulting in a much higher Ka/Ks ratio than in constitutive exons. The increased Ka values in alternative regions, at least in part, seem to be due to positive selection at the protein sequence level [50]. The cause of the low Ks values in ALT regions is unclear, but might have to do with more stringent requirements for RNA secondary structure in AS [49,51]. Here we did not observe this paradoxical relationship between Ka and Ks in ALT and CONSTIT uORFs in mammalian 5'UTRs (even after removing short uORFs from the calculation), but instead found a consistent pattern for all measured rates (Table 6). The likely explanation for this apparent difference between the evolution of alternative regions in 5'UTRs and CDS is that for uORFs, purifying selection at the amino acid level is weak at best and there is no positive selection; in contrast, selection for RNA secondary structure would almost equally affect Ka and Ks.

### Tight regulation of expression in genes with alternative 5'UTRs

We characterized the biological roles of genes that contain alternative regions in 5'UTRs, by examining patterns of gene expression and functional annotation for genes and transcripts within the ALT_5'UTR sets. Gene expression patterns for ALT_5'UTRs were evaluated using Atlas2 microarray and expressed sequence tag (EST) data. Gene Ontology annotation was used to classify ALT_5'UTRs according to biological process, molecular function and cellular localization patterns.

Average probe expression levels were calculated using probe data for the human genes in the analyzed set. Atlas2 expression data were separated into two groups: the ALT_5'UTR set (2097 probes) and the nonALT CONTROL set (8060 probes). We calculated average probe expression levels across the 79 tissue types in human and found that transcripts from the ALT_5'UTR set were expressed at significantly lower levels, on average, than transcripts from the nonALT CONTROL set (P = 7.3 × 10^-13^; Student's t-test). Furthermore, when sets of 1611 ALT and nonALT transcripts with size-matched 5'UTRs were compared, the statistically significant difference remained (P = 5.0 × 10^-5^; Student's t-test). This observation indicates that the difference in expression levels could not be explained, simply, by the greater average length of the 5'UTRs in the ALT_5'UTR set.

Average probe expression levels were also calculated using EST abundance as a measure of expression. The number of gene-specific EST sequences in the EST databases gives a reasonably accurate approximation of relative gene expression [52]. Alignments of transcript sequences from ALT_5'UTR and nonALT CONTROL sets with ESTs from the human normal tissue GenBank EST libraries were selected for analysis using thresholds given in the Methods section. Gene expression levels based on the analysis of EST database were calculated for 57 different human tissues. Significant differences between the EST data for ALT_5'UTR and nonALT CONTROL sets was demonstrated with a Monte Carlo approximation of Fisher's exact test (P < 10^-5^); compatible results were obtained with sets of 5733 transcripts with length-matched 5'UTRs (P < 10^-3^). Thus, the analysis of EST abundance data confirmed that transcripts from the ALT_5'UTR set that contain alternative regions in 5'UTRs are expressed at lower levels, on average, than transcripts from the nonALT CONTROL set, and the difference cannot be explained solely through the length differences between the 5'UTRs.

We classified ALT_5'UTR genes according to their biological roles, by searching for evidence of keyword enrichment from Gene Ontology annotation. The results suggest that the ALT_5'UTR set is enriched for genes that are strongly and tightly regulated. Human and mouse genes with alternative 5'UTRs are significantly enriched for keywords associated with biological functions such as signal transduction, receptor activity and translation (Table 7 and Additional file 2). This subset of genes also includes a large fraction of growth factors and transcription factors, which are known to be finely and strongly regulated [53,54]. Furthermore, in accordance with this observation, we found that over 17% of the annotated genes in our data set of human genes with alternative events in the 5'UTRs are classified as "precursor" proteins, which is compatible with the tight regulation of protein expression in this set of genes.

**Table 7:** Functional classification of human genes with alternative 5'UTRs

**GO keyword**	**ALT**	**ALL**	**P**
response to stimulus	20	597	4.2E-92
G-protein coupled receptor protein signaling pathway	52	842	1.8E-64
signal transduction	232	1778	1.2E-20
receptor activity	189	1425	1.3E-15
RNA binding	66	559	7.5E-11
membrane	754	4608	1.8E-09
translation	26	261	4.5E-09
protein folding	24	243	1E-08
extracellular space	59	480	2.7E-08
rhodopsin-like receptor activity	28	267	3.6E-08
extracellular region	92	686	7.3E-08
biological process	83	627	9.8E-08
nucleus	669	4043	2.1E-07
integral to membrane	571	3467	6.4E-07
DNA binding	195	1285	1.5E-06
nucleic acid binding	93	669	1.7E-06
proteinaceous extracellular matrix	23	210	5.1E-06
intracellular	303	1896	7.2E-06
RNA splicing	22	201	7.9E-06
regulation of apoptosis	33	101	2.1E-05

Human genes are partitioned into two groups: genes with alternative 5'UTRs (ALT) and ALL genes. Gene Ontology keyword descriptions are listed in left column. Keyword frequencies were tabulated for the ALT and ALL sets, and normalized by the total numbers in each set. P-values were calculated using the χ^2 ^test.

Qualitatively, the two groups of observations, those on the lower expression level of genes containing alternative regions in 5'UTRs and those on the tight regulation of the corresponding genes, appear congruent and compatible with plausible hypothesis that these genes are subject to especially strong down-regulation at both levels, transcription and translation, with the control elements in the alternative regions involved in the latter. This hypothesis is strengthened by results from a recent study which show that uORF-containing transcripts, on average, are expressed at lower levels and have shorter half-lives than transcripts without uORFs [55]. Thus, the effects of translational repression in uORF-containing transcripts, in part, might be achieved via an RNA decay mechanism.

## Conclusion

All findings presented here seem to be consistent with the hypothesis that alternative events, namely, AS and ATI, in 5'UTRs of mammalian genes contribute to the regulation of translation. At least within the set of genes that was conservatively defined to include genes with reliably demonstrated alternative events within 5'UTRs, the fraction of the 5'UTRs that is involved in an alternative event is much greater than that in the corresponding coding regions. In retrospect, this finding might not be particularly unexpected considering that 5'UTRs are not bound by constraints on protein structure and function, which limit the number of alternative nucleotides that are admissible in coding regions. The ratio of alternative-to-constitutive nucleotides is much higher in 5'UTRs than in coding regions, suggesting the possibility that alternative regions play a major role in regulating translation of the respective genes. With regard to this hypothesis, the results of the present analysis are somewhat ambiguous. The alternative regions of mammalian 5'UTRs contain a greater density of potential control elements (uAUGs and uORFs) than constitutive regions, but these elements are less conserved than those in constitutive regions. Thus, the fraction of conserved control elements among the uORFs contained within the alternative regions of mammalian 5'UTRs is lower than that in constitutive regions. Nevertheless, the uORF sequences even in alternative regions are, on average, subject to a stronger selection than the rest of the sequence. Furthermore, there is anecdotal experimental evidence that implicates non-conserved uORFs in translation regulation. Therefore, such variable elements provide considerable potential for regulation that, however, can be adequately explored only by direct experimentation.

The genes containing alternative regions in the 5'UTRs are relatively lowly expressed and typically belong to functional categories such as transcription factors, receptors, and other signaling pathway molecules, whose expression is strongly and tightly controlled. Together with the observations on the patterns of evolution of potential regulatory elements, these trends indicate that AS and ATI are probably important mechanisms of translation regulation in mammals.

## Methods

### The 5'UTR data set

Alternative splicing data were obtained from the ASD Database for human (Ensembl v36.35i) and mouse (Ensembl v37.34e) [56]. Those ASD isoforms that were generated on the basis of computational predictions and/or EST evidence alone were discarded, in effort to eliminate potentially unreliable splice predictions and alignment artifacts from biasing the results. In order to be included in the analysis, isoforms had to meet the following criteria: 1) isoforms must have supporting mRNA evidence, and 2) start and stop codon positions for coding sequences (CDS) must be known. Start codon positions were used to identify the subset of isoforms that included 5'UTR|CDS boundaries. The 7735 human transcripts selected by these criteria were used to identify 2915 human genes on the basis of the ASD information, and 2165 mouse transcripts were similarly used to identify 909 mouse genes. This filtering method underestimates the number of genes with alternative regions in 5'UTRs but yields a high quality dataset based upon the alignment of full-length mRNAs with protein and start codon annotations. We refer to these sets of human and mouse genes as the ALT_5'UTR sets, because the 5'UTRs of these genes contain alternative (ALT) and constitutive (CONSTIT) nucleotides. By definition, alternative nucleotides are present in a fraction of the transcripts of the respective genes, whereas constitutive nucleotides are present in all of the genes transcripts.

### Determination of the boundaries of 5'UTR and CDS

The 5'UTR and CDS boundaries for genes in the ALT_5'UTR set were determined by combining start and stop codon annotations from all isoforms that mapped to a gene. The 5'UTR boundaries for each gene were determined as follows: the upstream 5'UTR boundary was selected by choosing the most upstream isoform position; the downstream 5'UTR boundary was selected by choosing the most upstream start codon position from the set of isoforms that map to the gene (if more than one authentic start codon exists among the set of isoforms, the most upstream start codon position is chosen) (Figure 1). These conservative criteria ensure that protein-coding regions are excluded from the analysis of 5'UTR sequence variability. The exclusion of fragmentary sequence data is especially important in identifying the upstream 5'UTR boundary, as short non-coding RNA sequences are often positioned immediately upstream of the 5'-end of coding genes [57].

The CDS boundaries were determined as follows: the upstream CDS boundary was selected by choosing the most upstream start codon position from the set of isoforms; the downstream CDS boundary was selected by choosing the most upstream stop codon position from the set of isoforms that map to the gene (if more than one authentic stop codon exists, the most upstream stop codon position is chosen (Figure 1).

### Detection of potential regulatory elements in mammalian 5'UTRs

Starting with 5'UTR leader sequences from the isoforms in the ALT_5'UTR set, we calculated the uAUG and uORF abundance in ALT and CONSTIT regions of these 5'UTR sequences. The uAUGs were identified by searching for AUG triplets within the 5'UTRs. The uAUGs were then mapped to the genomic sequence, where redundant elements (uAUGs from different isoforms that mapped to the same genomic coordinates) were removed from the final set. The uORFs were also detected in the 5'UTRs and similarly mapped to the genomic sequence. The uORFs were identified using the EMBOSS program getorf [58], with the following criteria: 1) uORFs must be at least 6 nucleotides in length, 2) a uORF must start with methionine and end with one of the three stop codons, and 3) uORFs are identified in all reading frames.

uAUGs and uORFs were classified as ALT or CONSTIT based upon their overlap with ALT and CONSTIT regions of the corresponding genomic sequence. Only uAUGs and uORFs that are fully contained within ALT and CONSTIT regions of 5'UTRs were included in this study (i.e., uAUGs and uORFs that overlapped between constitutive and alternative regions were excluded).

### Identification of orthologous genes

Human-macaque (human version NCBI36; macaque version MMUL1) and mouse-rat (mouse version NCBI36; rat version RGSC3.4) orthologous gene pairs were downloaded from Ensembl using the BioMart data mining tool [59]. 5'UTR alignments were generated using the OWEN alignment tool [60] with the following parameters: a P-value < 0.001 for each hit was required, and 5'UTRs were required to be bound at the 3' ends by exons that align across > 80% of length [61]. For the beginning of the CDS, alignment of the nucleotide sequences was guided by the amino acid sequence alignment [62]. Putative ortholog alignments were cleaned using previously reported thresholds [63]. In cases where greater than 40% of the gaps or unannotated regions of orthologous sequences did not align, the orthologs were removed from the final set. For example, 5'UTRs of many macaque genes are not properly annotated (contain 'NNNNN'), making it difficult to identify the upstream 5'UTR boundary. The human-macaque alignments with uncertainties of this type were discarded. In all, in a total of ~2800 human-macaque and ~900 mouse-rat whole gene alignments were generated.

### Comparison of substitution rates in coding and non-coding DNA

Synonymous (Ks) and non-synonymous (Ka) substitution rates for the uORFs located in the 5'UTR were calculated using the Pamilo-Bianchi-Li method [64,65] which takes into account transition and transversion rates. Rates of divergence were calculated separately for uORFs located in constitutive and alternative regions of 5'UTR. We calculated Ka and Ks separately for all uORFs and for the subset of uORFs ≥ 30 nucleotides in length, to ensure that short uORFs did not bias the results. Substitution rates for the 5'-untranslated regions with uORFs (K_5 uORFs_) and without uORFs (K_5 uORFs excluded_) were calculated along the 5'UTRs for each gene using Kimura's two-parameter model [66].

### Gene expression analysis

GNF Atlas2 expression data was downloaded from the UCSC genome browser (gnfAtlas2 table) to study gene expression patterns in human (Mar.2004 assembly) and mouse (Aug.2005 assembly) [67,68]. GenBank mRNA accession IDs were used to map isoform sequences to probe data. Probe data were partitioned into two groups: the ALT_5'UTR sets, and the nonALT CONTROL sets, which contained the full complement of probe data. The nonALT CONTROL set consists of transcripts whose 5'UTRs have not been shown to undergo alternative events such as AS or ATI. Atlas2 expression data are classified into 79 distinct tissue types in human and 61 tissues types in mouse, and duplicate intensity values for each tissue type are included in the raw data. Replicates were combined by calculating the average intensity value for each tissue type. Average expression levels were calculated separately for individual probes and individual tissue types. Average probe expression levels were calculated by summing intensity values across all tissue types in the probe, and dividing by the total number of tissues. Average tissue expression levels were calculated by summing intensity values for a set of probes and dividing by the total number of probes in the dataset.

### Gene expression levels based on EST abundance

Gene expression levels were also evaluated by tallying the numbers of gene-specific EST sequences in the databases. Transcript sequences from the ALT_5'UTR set and the nonALT control set were aligned with ESTs from the human normal tissue GenBank EST libraries (~8 millions of ESTs, release 071808) using the BLASTN program [69]. EST hits with the identity more than 95% and longer than 80% of EST sequence length were accepted as matches. Gene expression levels based on EST abundance calculated for 57 normal human tissues [70] were used for statistical analysis. Similar tissue-specific preferences were considered for both the ALT_5'UTR and nonALT control sets in the final classification and statistical analysis. A Monte Carlo approximation of Fisher's exact test implemented in the COLLAPSE program [71] was used to assess the significance of the differences between the EST data for ALT_5'UTR and nonALT control sets.

### Gene Ontology Annotation

Functional annotation for human and mouse was downloaded from the Gene Ontology (GO) database [72]. Starting with a total of 16,468 annotated human genes, GO annotations were mapped to 89% (2608) of the genes in the ALT_5'UTR set. With 17,480 annotated mouse genes, GO annotations were mapped to 94% (861) of the genes in our ALT_5'UTR set. Keyword frequencies were tabulated for the ALT and ALL sets, and normalized by the total numbers in each set. P-values were calculated using the χ^2 ^test.

## Competing interests

The authors declare that they have no competing interests.

## Authors' contributions

AMS contributed to the design of the study, performed the bulk of the data analysis and wrote the initial draft of the manuscript, AYO contributed to data analysis, IBR contributed to the design of the study and data analysis, SAS contributed to the design of the study and data analysis, EVK contributed to the design of the study and wrote the final version of the manuscript; all authors read and approved the final version.

## Supplementary Material

Additional file 1 **Supplementary Figures**. Additional figures and controls for the analysis of alternative and constitutive 5'UTRs. Click here for file

Additional file 2 **Supplementary Tables**. Additional tables and comments on the analysis. Click here for file
