# Supplementary material for: Evolution of alternative and constitutive regions of mammalian 5'UTRs
Source: BMC Genomics. 2009 Apr 16;10:162. doi: 10.1186/1471-2164-10-162 (PMC2674463; doi:10.1186/1471-2164-10-162)

**Supplementary Figure Legends**

**Figure S1**

**5’UTR length distributions for ALT and nonALT gene sets**. Comparison of 5’UTR length distributions for transcripts in ALT (blue) and nonALT control (red) sets in human (top) and mouse (bottom). Genes in ALT set exhibit 5’UTR transcript diversity, whereas genes in nonALT set do not. 5’UTR lengths were partitioned into 30 nucleotide bins; counts for lengths greater than 930 nucleotides are combined in the last bin. Significant differences in 5’UTR length distributions between the ALT and nonALT control sets were observed in human (P = 5.8x10^-5^; Mann-Whitney test), but not mouse (P=0.50; Mann-Whitney test).


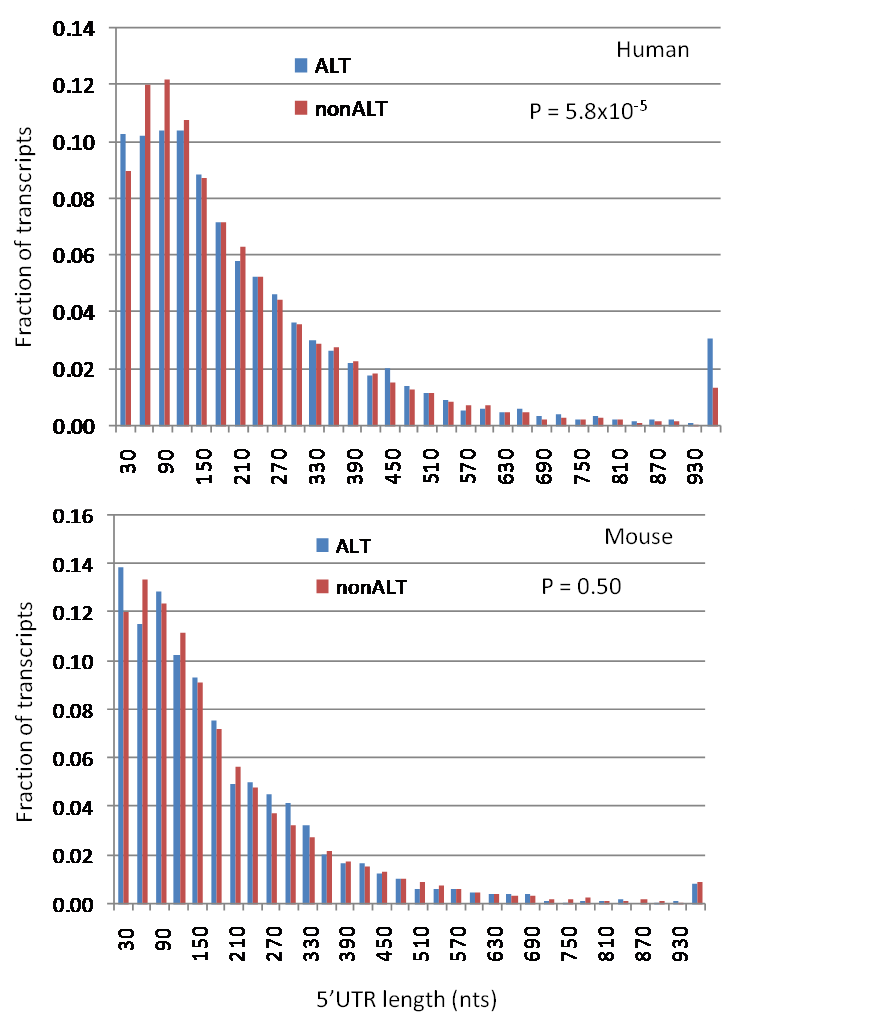


**Figure S2**

**Codon frequencies for alternative and constitutive 5’UTRs**. Relative codon frequencies for alternative (blue) and constitutive (red) regions of 5’UTR in human.


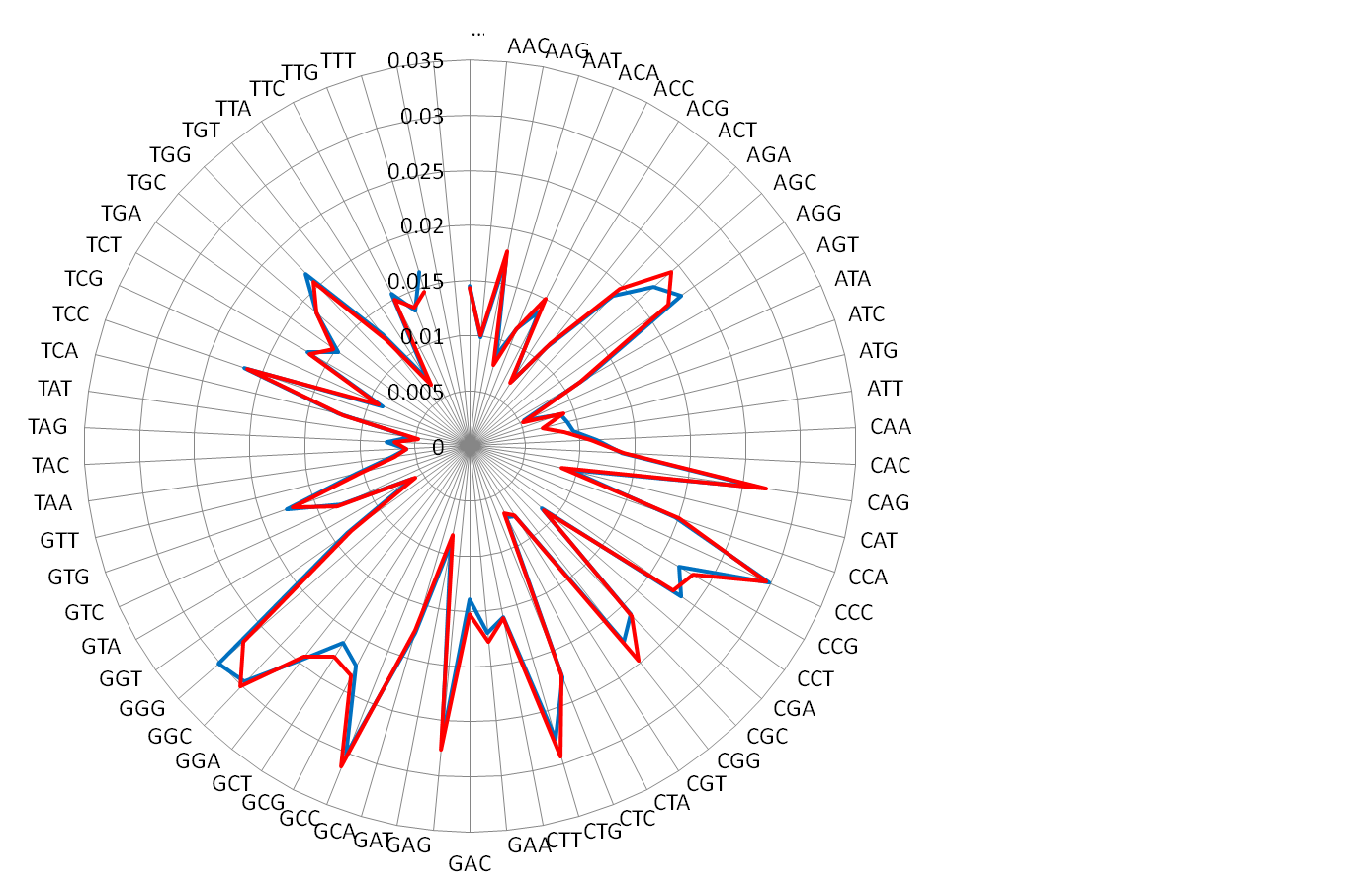


**Figure S3**

**uORF length distributions for size-matched alternative and constitutive regions**. uORF length distributions for size-matched alternative (blue) and constitutive (gray) regions in human. Genes with size-matched ALT and CONSTIT regions were identified by calculating the absolute values between the ALT length and CONSTIT length of each gene and selecting the subset of genes where the absolute value |ALT_length – CONSTIT_length| ≤ 0.30. uORF lengths are grouped into bins of 15 nucleotides each, and uORFs greater than 420 nucleotides are grouped together in the last bin. uORF lengths are distributed differently between ALT and CONSTIT regions (P = 0.00006; Student’s t-test) .


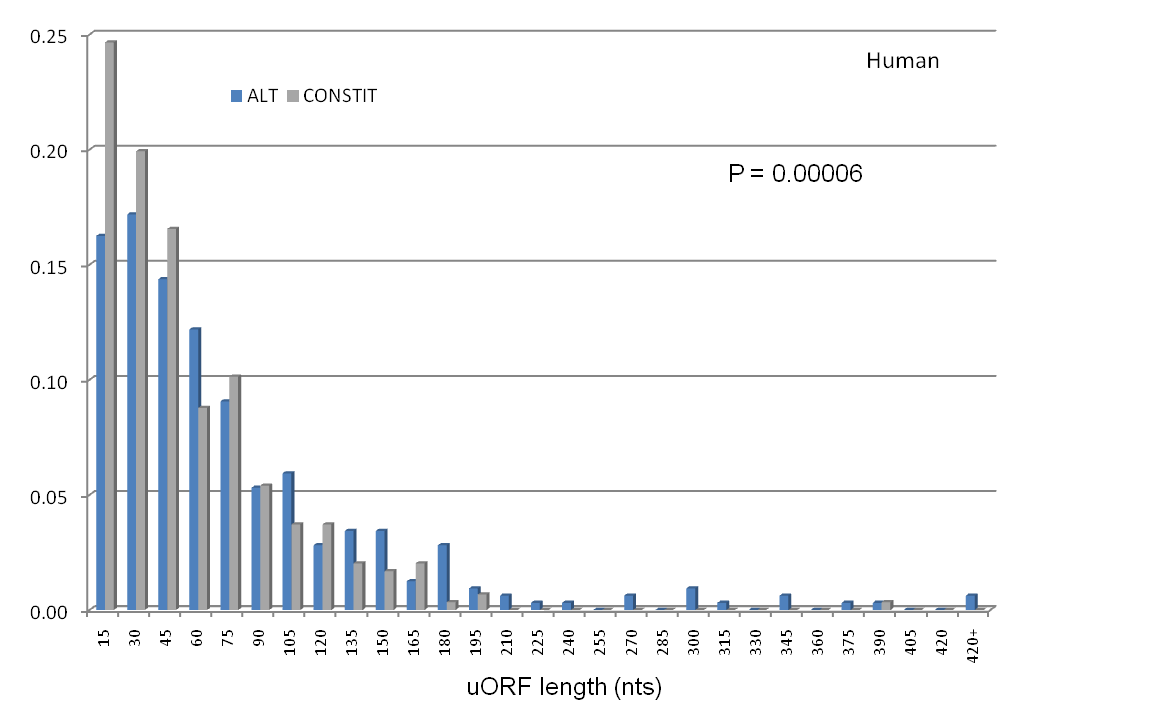


**Figure S4**

**uORF density in ALT and nonALT gene sets**. uORF density (number of uORFs per gene) in genes from ALT (blue) and nonALT control (red) genes in human (top) and mouse (bottom). uORF densities are significantly different between genes in the ALT and nonALT control sets in both human (P=1.46x10^-11^; Student’ t-test) and mouse (P=0.0004; Student’s t-test).


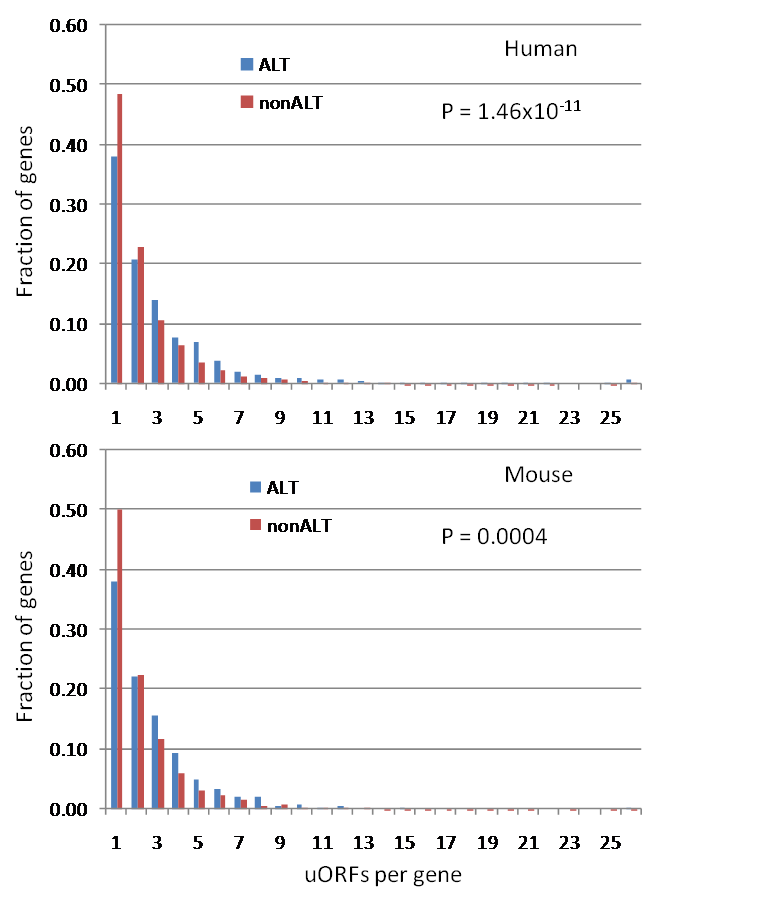


**Figure S5**

**uORF length distribution in nonALT control sets**. uORF lengths (nts) for genes from the nonALT control sets in human (top) and mouse (bottom). uORF lengths are partitioned into 30 nucleotide bins; counts for lengths greater than 570 nucleotides are combined in the last bin.


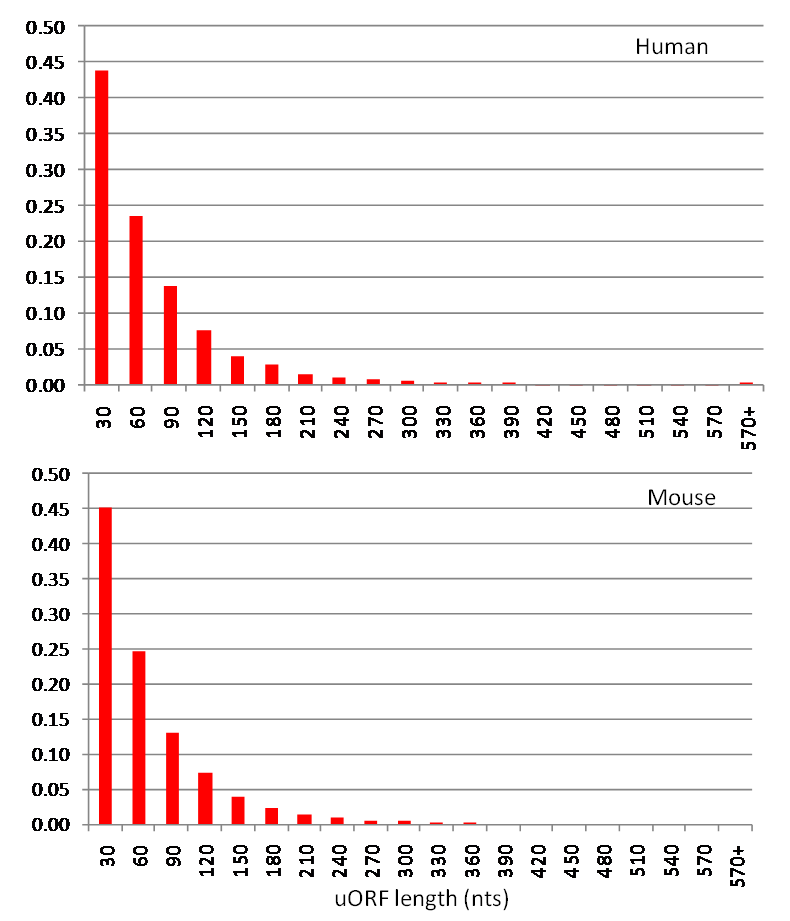


**Figure S6**

**Codon frequencies for alternative and constitutive uORFs**. Codon frequencies for uORFs located in ALT (blue) and CONSTIT (red) regions of 5’UTR in human.


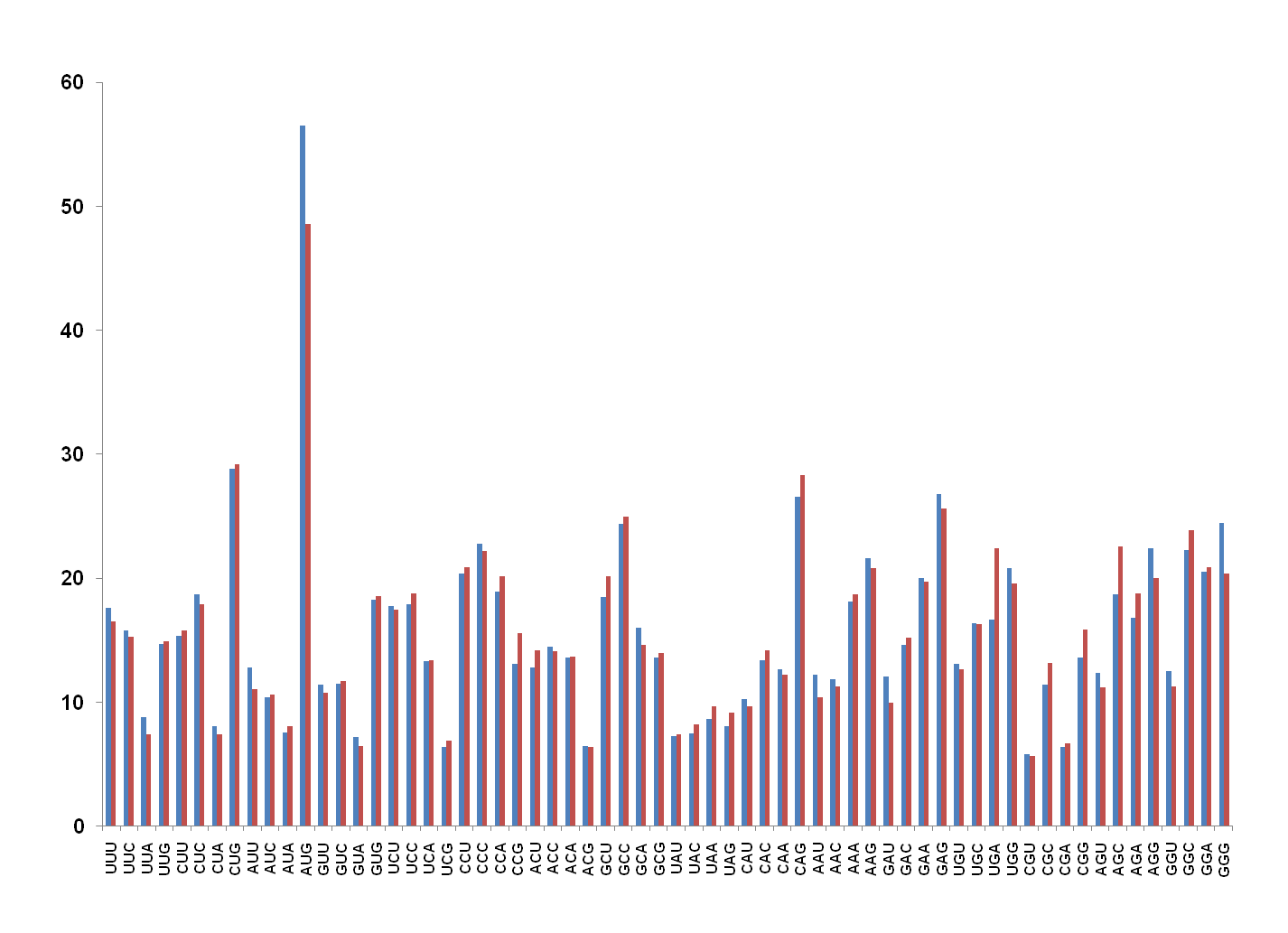

Supplement: Additional file 1 — Supplementary Figures. Additional figures and controls for the analysis of alternative and constitutive 5'UTRs. [file 1471-2164-10-162-S1.doc]
