# Supplementary material for: Evolution of alternative and constitutive regions of mammalian 5'UTRs
Source: BMC Genomics. 2009 Apr 16;10:162. doi: 10.1186/1471-2164-10-162 (PMC2674463; doi:10.1186/1471-2164-10-162)
Supplement: Additional file 2 — Supplementary Tables. Additional tables and comments on the analysis. [file 1471-2164-10-162-S2.doc]

**SUPPLEMENTARY TABLES**

**Table S1**: **Frequency of A, T, G and C nucleotides in the -3 position upstream of conserved uAUGs**

|  | **Hum-Mac** |  |  | **Mm-Rat** |  |  |
| --- | --- | --- | --- | --- | --- | --- |
|  | **ALT** | **CONSTIT** | **SC** | **ALT** | **CONSTIT** | **SC** |
| **A** | 0.27 | 0.24 | 0.5 | 0.25 | 0.24 | 0.53 |
| **T** | 0.23 | 0.26 | 0.03 | 0.24 | 0.26 | 0.04 |
| **G** | 0.28 | 0.29 | 0.4 | 0.3 | 0.26 | 0.36 |
| **C** | 0.22 | 0.21 | 0.07 | 0.21 | 0.24 | 0.07 |

Percentage of A,T,G and C nucleotides in the -3 position for the set of conserved uAUGs located in alternative (ALT) and constitutive (CONSTIT) regions of 5’UTR, upstream of the authentic start codon (SC) for human-macaque and mouse-rat.

**Table S2**: **Nucleotide composition in alternative and constitutive regions of 5’UTR**

|  | **Human** |  | **Mouse** |  |
| --- | --- | --- | --- | --- |
|  | **ALT** | **CONSTIT** | **ALT** | **CONSTIT** |
| **A** | 0.2 | 0.2 | 0.2 | 0.21 |
| **T** | 0.2 | 0.2 | 0.21 | 0.2 |
| **G** | 0.31 | 0.31 | 0.3 | 0.29 |
| **C** | 0.29 | 0.29 | 0.29 | 0.3 |

Percentage of A, T, G and C nucleotides in alternative (ALT) and constitutive (CONSTIT) regions of human and mouse 5’UTRs.

**Table S3**: **Functional classification of mouse genes with alternative 5’UTRs**

| **GO keyword** | **ALT** | **ALL** | **P** |
| --- | --- | --- | --- |
| G-protein coupled receptor protein signaling pathway | 28 | 1756 | 2E-129 |
| signal transduction | 86 | 2286 | 4.6E-41 |
| receptor activity | 101 | 2556 | 8E-40 |
| mitochondrion | 20 | 809 | 4.7E-35 |
| integral to membrane | 253 | 5016 | 2.2E-29 |
| rhodopsin-like receptor activity | 11 | 526 | 2.3E-29 |
| membrane | 287 | 5471 | 1.3E-25 |
| translation | 8 | 372 | 1.6E-20 |
| proteolysis | 19 | 582 | 6.5E-16 |
| peptidase activity | 15 | 488 | 3E-15 |
| protein-tyrosine kinase activity | 43 | 303 | 2.2E-14 |
| GTP-binding | 9 | 333 | 1.8E-13 |
| ribonucleoprotein complex | 8 | 304 | 6.2E-13 |
| intracellular | 92 | 1836 | 4.1E-12 |
| androgen receptor signaling pathway | 8 | 25 | 4.1E-10 |
| oxidoreductase activity | 25 | 617 | 4.4E-10 |
| protein kinase activity | 54 | 473 | 7E-10 |
| protein serine/threonine kinase activity | 54 | 474 | 8.1E-10 |
| zinc ion binding | 102 | 1935 | 9E-10 |
| iron ion binding | 10 | 304 | 7E-09 |

Mouse genes are partitioned into two groups: genes with alternative 5’UTR regions (ALT) and ALL genes. Gene Ontology keyword descriptions are listed in left column. Keyword frequencies were tabulated for the ALT and ALL sets, and normalized by the total numbers in each set. P-values were calculated using the χ^2^ test.

**SUPPLEMENTARY MATERIAL**

**Additional control**

Approximately 35% of human and 27% of mouse genes from the ALT_5’UTR set contain ALT nucleotides, but not CONSTIT nucleotides. To prevent this natural bias from skewing the results, we removed genes whose 5’UTRs lack CONSTIT nucleotides and recalculated uAUG frequencies in ALT and CONSTIT regions within the subset of genes whose 5’UTRs contain both ALT and CONSTIT nucleotides. Our analysis showed that even after removing the genes that lack CONSTIT nucleotides, uAUGs are 1.4 times more abundant in ALT versus CONSTIT regions in human and 1.3 times more abundant in mouse. Moreover, uAUGs within this set appear to be distributed differently between ALT and CONSTIT regions in human (P = 8x10^-24^; Students’s t-test) and mouse (P = 3.3x10^-7^; Student’s t-test). The data also showed that uORFs are 1.3 times more abundant in ALT regions in human and 1.2 times more abundant in mouse. There is a significant difference in the distribution of uORFs between ALT and CONSTIT regions in human (P = 3x10^-27^; Student’s t-test) and mouse (P = 1.4x10^-8^; Student’s t-test) within the subset of genes that contain ALT and CONSTIT nucleotides.
